# Agreement between self-/home-measured and assessor-measured waist circumference at three sites in adolescents/children

**DOI:** 10.1371/journal.pone.0193355

**Published:** 2018-03-22

**Authors:** Noel Po Tai Chan, Marie Tarrant, Esther Ngan, Hung Kwan So, Kris Yuet Wan Lok, Edmund Anthony Severn Nelson

**Affiliations:** 1 School of Nursing, The University of Hong Kong, Hong Kong, SAR, China; 2 School of Nursing, The University of British Columbia, Okanagan, Canada; 3 Biostatistics and Clinical Research Methodology Unit, The University of Hong Kong, Hong Kong, SAR, China; 4 Department of Paediatrics, The Chinese University of Hong Kong, Hong Kong, SAR, China; UT School of Public Health, UNITED STATES

## Abstract

The objective of this study was to assess the validity of the self-/home-measured waist circumference (WC) method in children/adolescents at three sites: at the level of the umbilicus, immediately above the iliac crest, and at the midpoint of the lower margin of the last palpable rib and top of the iliac crest. A cross-sectional study of 3360 Hong Kong Chinese children/adolescents was conducted, with 2980 (88.7%) participants included in the final analysis. The WC of children aged 6 to 9 was measured at the three sites by their parent/guardian at home followed by measurement by trained assessors at school within one week. Children/adolescents between the ages of 10 and 17 self-measured their WC at the three sites during school hours, followed by measurements by the trained assessors. Bland-Altman limits of agreement (LOA) analysis was performed to evaluate between-measurement agreement. The difference between assessor- and self-/home-measured WC was defined as ≤ ±2.5 cm for the upper and lower LOA at all three sites as an a priori criterion based on the assessor-measured inter-rater results. The results showed that most measurements (about 96%) at each site was within 95% of the LOA. Of the three measurement sites, the smallest LOA interval width was found at the umbilicus site, with an upper LOA of 5.08 and 7.13 and lower LOA of -2.61 and -3.43 in boys and girls, respectively. In conclusion, the range of LOA was relatively large, exceeding the acceptable limits of the predefined a priori criterion of upper and lower LOA, and thus suggesting disagreement between the two measurement methods. The use of WC as a measure of abdominal obesity in clinical practice/epidemiological studies should be restricted to measurement by trained health professionals/research staff.

## Introduction

The incidence of overweight and obesity (defined as weight exceeding 120% of the median weight for height) in Hong Kong children/adolescents aged 6 to 18 years was about 20% in 2013–2014 [1]. Children/adolescents with general obesity (as measured by weight for height ‘*squared’*) or abdominal obesity (as measured by waist circumference [WC]) are at increased risk of developing type 2 diabetes and cardiovascular disease early in life [2]. WC, an inexpensive, non-invasive anthropometric measure, has been recommended as a surrogate marker for assessing abdominal fat, including subcutaneous and visceral adipose tissue [3–5], and obesity-associated cardiometabolic risk [6] in both epidemiological studies [7, 8] and clinical practice.

The rising trend in childhood obesity has led to the need for a reliable WC self-measurement protocol for identifying and monitoring changes in a child’s abdominal obesity status. The common WC measurement sites in clinical settings and epidemiological studies are at the midpoint of the lower rib and top of the iliac crest [4], at the top of the iliac crest [5], and at the level of the umbilicus [9]. To the best of our knowledge, few studies to date have sought to validate self- and home-measured WC in adolescents and young children, respectively, at any of these three WC measurement sites, although self-reported WC in adolescents/children has been found to correlate with assessor-measured WC using intra-class correlation (ICC) statistics without predefined benchmark locations for the WC measurement site [10]. The self-reported WC is also associated with higher measurement error compared to assessor-measured WC [11]. In the remainder of the paper, both adolescents’ self-measured and young children’s home-measured WC are referred to as self-measured WC. The objectives of the study reported herein were to assess the agreement between self/home-measured WC and assessor-measured WC at three WC sites, namely, at the level of the umbilicus (WC1) [9], immediately above the iliac crest (WC2) [5], and at the midpoint of the lower margin of the last palpable rib and top of the iliac crest (WC3) [4].

## Materials and methods

### Study design

A cross-sectional study using a convenience sample was conducted in 2013–2014. Children/adolescents were recruited from six primary and four secondary schools from the three main regions of Hong Kong (Hong Kong Island [one primary and one secondary school], Kowloon [three primary and two secondary schools], and the New Territories [two primary and one secondary schools]). All students in the 10 schools were invited to participate in the study, and all were of Hong Kong Chinese descent.

#### Sample population

A total of 3360 students aged 5 to 22 years old were initially recruited for this study, of whom 1464 (43.6%) were primary school students and 1896 (56.4%) were secondary school students. Participants under the age of 6 or over the age of 17, as well as those with missing data (e.g., height, weight, any of the three WC measurements, birth year), errors, and/or WC outliers, were excluded. WC outliers were defined as a difference between two repeated measurements that was greater than three standard deviations. The final sample thus comprised 2980 (88.7%) participants (1616 boys [54.2%] and 1364 girls [45.8%]) eligible for data analysis.

#### Procedures

The WC of children in grades 1 to 3 (approximately 6- to 9-years-old) was measured at home by a parent/guardian/carer after a pilot test assessing the capacity of this age group for WC self-measurement. The children were given a non-elastic tape measure similar to that used in home sewing, a standard WC measurement protocol for the three WC sites, and a self-administered questionnaire that included demographic information to be completed at home. Their parents/guardians were asked to measure the children’s WC at the three sites before bedtime. The children were asked to return the completed questionnaire before or on the day of the WC measurements by the trained assessors. The trained assessors then measured the children’s WC at school within one week of their home-measured WC being recorded.

The older children, those in grade 4 or above, were asked to self-measure their WC at the three sites at school using the same WC measurement instructions supplied to the parents/guardians of the younger children. The trained assessors then measured them at school in private, with the girls being measured by female assessors. Data were collected during physical education lessons or free class time to minimize the disturbance to classroom learning activities.

To ensure a high degree of inter-rater agreement between assessors, prior to data collection, the assessors (n = 6) were asked to review the three standard WC measurement protocols to be used in the study, and they then practiced taking WC measurements until they had achieved a high degree of accuracy in comparison with one of the investigators who is experienced in WC measurement before performing interrater agreement assessment between assessors. As there is no guideline on the acceptable degree of between-assessor discrepancy for WC measurement [12], the inter-rater agreement assessment data were then examined using Bland-Altman limits of agreement (LOA) analysis (n = 70). The results revealed no statistically significant mean differences (MD) between the assessors and the investigator for any of the measurement sites (WC1: MD = -0.08 (95% confidence interval [CI]: -0.34, 0.18); WC2: 0.04 (-0.25, 0.34); WC3: -0.2 (-0.44, 0.03)]. The width of the LOA interval was narrow, with an upper and lower LOA of less than ± 2.46 cm at the three WC measurement sites (ranging from 1.74 to 2.46). LOA of about ≤ ± 2.5 cm was considered indicative of acceptable limits of agreement between the two measurement methods.

For illustrative purposes, inter-rater reliability was also analyzed to determine the strength of the relationship between the two measurement methods using ICC statistics. Reliability is defined as the extent to which measurements can be replicated [13]. The results showed excellent inter-rater reliability (ICC values ranging from 0.93–0.97; n = 70).

#### Anthropometric measurement methods and definitions

Waist circumference: The participants were asked to stand in a relaxed, straight, and upright position with their feet slightly apart. A flexible but non-elastic tape measure was fitted snugly to participants’ bare skin on a horizontal plane around the abdomen without causing any indentation. The WC (cm) at each site (WC1 to WC3) was measured to the nearest 0.1 cm at the end of normal expiration of the breath. Two measurements were taken at each site by both trained assessors and participants using the same instructions and same type of tape measure throughout the study, with the measurements then averaged for data analysis.

Body height (BH) and body weight (BW): Standing height (m) was measured without shoes to the nearest 0.1 cm using a portable stadiometer (seca 213), and weight (kg) was measured to the nearest 0.1 kg by a digital scale (Tanita model number TBF 410), with the participants wearing light clothing. Both height and weight were measured twice, with the two measurements then averaged for data analysis. BMI was calculated by weight in kg divided by the square of height in meters (m^2^).

Anthropometric cutoff for children/adolescents aged 6–17 years: The BMI cutoff for children/adolescents aged 6–17 was as follows.

- Weight status defined by age- and sex-specific BMI
  - Overweight: ≥ 85^th^ and < 95^th^ percentiles
  - Obese: ≥ 95^th^ percentile
  - Underweight: ≤ 5^th^ percentile

The above cutoff values are based on studies conducted on Hong Kong school children [14–16].

### Ethical approval

Ethical approval was obtained from the Institutional Review Board of the University of Hong Kong/Hospital Authority Hong Kong West Cluster and the administrators of all participating schools. Informed written consent was obtained from the participating parents and children through the schools. Confidentiality was ensured, and the children were informed that they could withdraw from the study at any time without penalty.

### Statistical analysis

Data were summarized, and are presented herein, using appropriate descriptive statistics, e.g., mean ± standard deviations (SD) and mean differences (± 95% CI). The differences between the assessor-measured WC and self-measured WC values in the subgroups of participants were compared using a two-sample t-test. The average agreement between the two in each WC site was assessed via Bland-Altman analysis [17], and bias was evaluated by the mean difference. In theory, the mean difference should be zero because the same WC site was used in both assessor-measured WC and self-measured WC. The mean difference refers to the mean of the assessor-measured WC value minus the mean of the self-measured WC value, with a negative between-measurement difference indicating that the participant’s self-measured WC was larger than his or her assessor-measured WC. A positive difference, in contrast, indicated that the participant’s self-measured WC was smaller than his or her assessor-measured WC.

We defined the 95% LOA between assessor-measured WC and self-measured WC as within two SDs of the mean differences [18]. The 95% confidence limits were calculated as mean difference ± 1.96 multiplied by the SD of the differences. If the mean difference was not zero, then one measure was considered to be biased over the other. Points outside the limits were considered to be significant differences. There is no standard interpretation of the LOA range in Bland-Altman analysis. The basic principle is that the smaller the range of two limits, the better the agreement between two measures. Owing to a lack of guidance on inter-rater differences in WC measurement among trained health professionals, no standardized difference of agreement currently exists for classifying the validity between the two measurement methods. Thus, an a priori difference not exceeding ≤ ± 2.5 cm between assessor-measured WC and self-measured WC was defined as indicative of an acceptable agreement based on our inter-rater agreement results [17, 19]. The a priori difference in agreement was then compared with the LOA results.

The inter-rater reliability between assessor-measured WC and self-measured WC for each WC measurement site was also assessed via the ICC.

## Results

**Fig 1:**
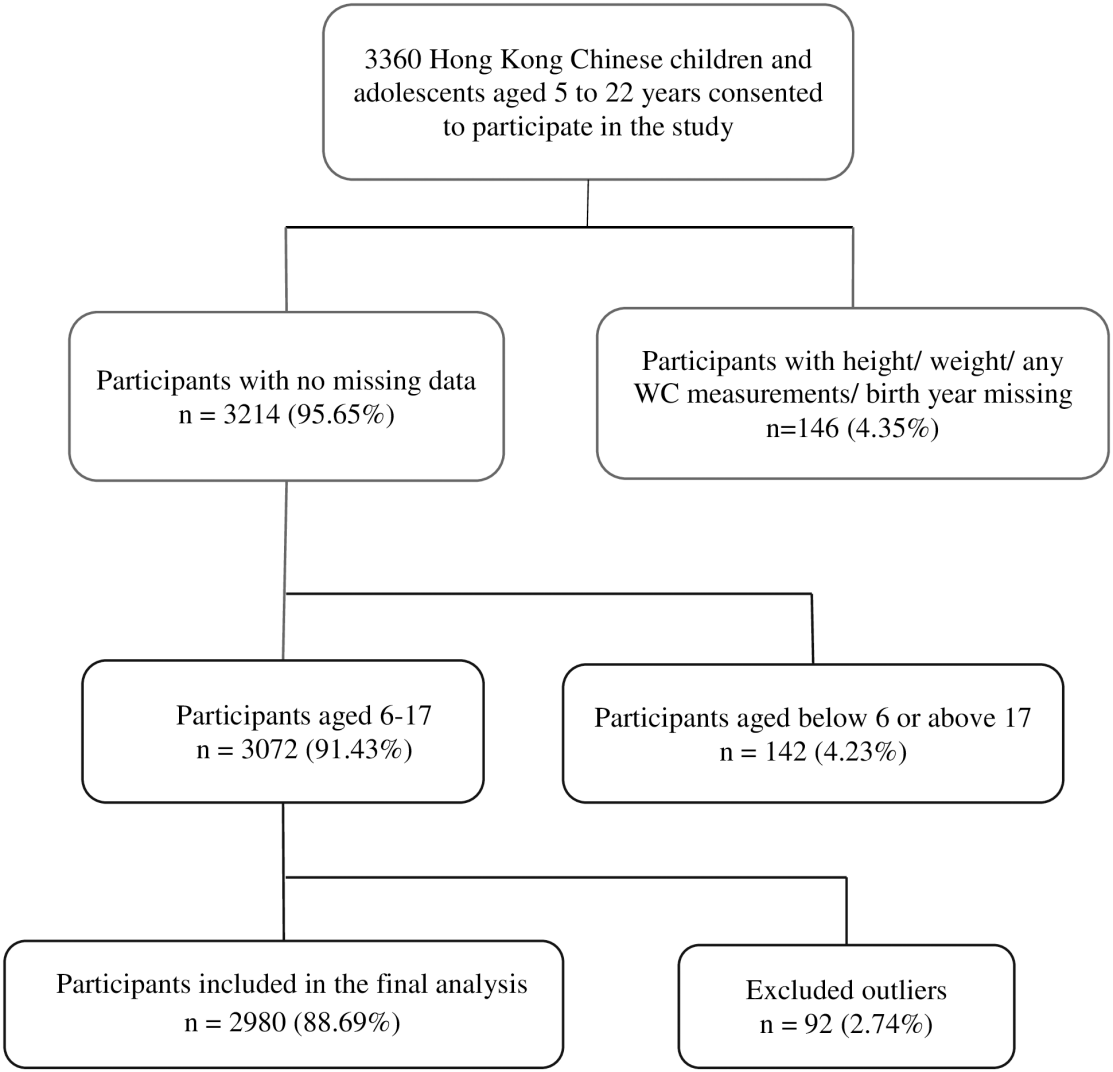
Recruitment of the study population aged 6 to 17 years.

An overview of the cohort of children/adolescents who were included in and excluded from the study is shown in Fig 1. The characteristics of the final 2980 children/adolescents participating in the study are described in Table 1. About one in four (25.9%) of the participants was found to be overweight or obese.

**Table 1:** Characteristics of eligible participants.

	All (n = 2980)	Boys (n = 1616)	Girls (n = 1364)	p-value (boys vs girls)
**Age (yrs)**	12.0 (3.3)	12.0 (3.3)	12.1 (3.3)	0.207
**BMI**	19.0 (3.8)	19.2 (4.0)	18.8 (3.5)	0.007
**Weight status:**				0.068
• **Underweight**	62 (2.1%)	29 (1.8%)	33 (2.4%)	
• **Normal**	2145 (72.0%)	1158 (71.7%)	987 (72.4%)	
• **Overweight**	439 (14.7%)	228 (14.1%)	211 (15.5%)	
• **Obese**	334 (11.2%)	201 (12.4%)	133 (9.8%)	

### Agreement between assessor-measured and self-measured waist circumference at 3 sites (Fig 2, Table 2, and S1, S2 & S3 Tables)

**Fig 2:**
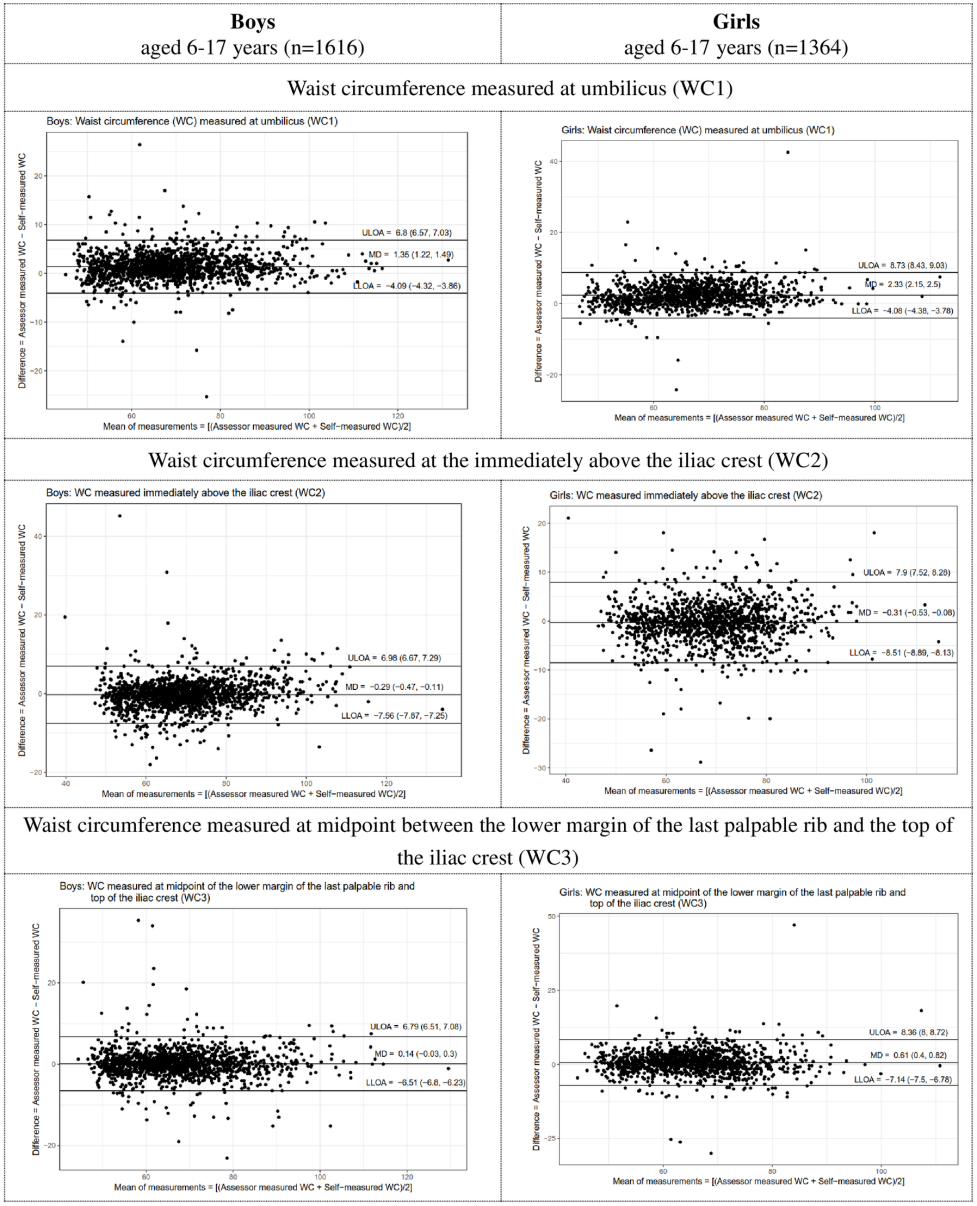
Bland altman plots illustrating the level of agreement between assessor-measured & self-measured WC at 3 WC measurement sites by sex and location. MD: Mean difference ULOA: Upper limits of agreement LLOA: Lower limits of agreement.

**Table 2:** Limits of agreement analysis of 3 waist circumference measurement sites between assessor-measured and home measured/self-measured values by age groups and sex.

	Boys					Girls			
Age		N	Bias (95% CI)	LLOA (95% CI)	ULOA (95% CI)	N	Bias (95% CI)	LLOA (95% CI)	ULOA (95% CI)
	Home-measured WC					Home-measured WC			
**6–7**	WC1	204	0.27 (-0.31, 0.84)	-7.87 (-8.85, -6.89)	8.40 (7.42, 9.39)	155	0.52 (-0.06, 1.10)	-6.66 (-7.65, -5.66)	7.70 (6.70, 8.69)
	WC2	204	-0.98 (-1.68, -0.28)	-10.91 (-12.1, -9.71)	8.95 (7.75, 10.15)	155	-1.56 (-2.34, -0.79)	-11.12 (-12.45, -9.80)	8.00 (6.67, 9.32)
	WC3	204	-0.50 (-0.76, 0.27)	-7.57 (-8.45, -6.69)	7.07 (6.19, 7.95)	155	-0.59 (-1.26, 0.09)	-8.91 (-10.06, -7.75)	7.63 (6.58, 8.89)
**8–9**	WC1	216	0.90 (0.55, 1.25)	-4.26 (-4.86, -3.66)	6.06 (5.45, 6.66)	183	1.24 (0.82, 1.66)	-4.45 (-5.17, -3.72)	6.92 (6.20, 7.65)
	WC2	216	-0.99 (-1.55, -0.42)	-9.22 (-10.18, -8.25)	7.24 (6.28, 8.21)	183	-0.81 (-1.39, -0.24)	-8.58 (-9.57, -7.59)	6.95 (5.96, 7.94)
	WC3	216	-0.60 (-1.09, -0.10)	-7.76 (-8.60, -6.93)	6.57 (5.73, 7.41)	183	-0.40 (-1.06, 0.27)	-9.37 (-10.52, -8.23)	8.58 (7.44, 9.73)
	Self-measured WC					Self-measured WC			
**10–11**	WC1	248	1.23 (0.99, 1.48)	-2.61 (-3.03, -2.19)	5.08 (4.66, 5.50)	239	1.85 (1.51, 2.19)	-3.43 (-4.01, -2.84)	7.13 (6.54, 7.71)
	WC2	248	0.02 (-0.34, 0.38)	-5.61 (-6.22, -4.99)	5.65 (5.04, 6.27)	239	0.09 (-0.40, 0.58)	-7.44 (-8.27, -6.60)	7.62 (6.78, 8.45)
	WC3	248	-0.15 (-0.48, 0.18)	-5.32 (-5.88, -4.75)	5.01 (4.45, 5.57)	239	0.46 (0.06, 0.86)	-5.68 (-6.36, -4.99)	6.59 (5.91, 7.27)
**12–13**	WC1	369	1.80 (1.52, 2.09)	-3.61 (-4.09, -3.12)	7.22 (6.73, 7.70)	245	2.97 (2.62, 3.33)	-2.59 (-3.21, -1.98)	8.54 (7.94, 9.15)
	WC2	369	0.10 (-0.24, 0.44)	-6.49 (-7.08, -5.90)	6.69 (6.10, 7.28)	245	0.39 (-0.13, 0.91)	-7.69 (-8.58, -6.81)	8.48 (7.59, 9.37)
	WC3	369	0.34 (-0.07, 0.75)	-7.52 (-8.22, -6.82)	8.20 (7.50, 8.90)	245	0.90 (0.50, 1.30)	-5.34 (-6.02, -4.65)	7.14 (6.45, 7.82)
**14–15**	WC1	296	1.55 (1.29, 1.82)	-3.02 (-3.48, -2.57)	6.13 (5.67, 6.59)	264	3.29 (2.82, 3.75)	-4.26 (-5.06, -3.46)	10.83 (10.04, 11.63)
	WC2	296	-0.10 (-0.50, 0.29)	-6.87 (-7.54, -6.19)	6.67 (5.99, 7.34)	264	0.03 (-0.46, 0.51)	-7.86 (-8.70, -7.03)	7.91 (7.08, 8.75)
	WC3	296	0.47 (0.19, 0.74)	-4.26 (-4.73, -3.78)	5.19 (4.72, 5.66)	264	1.16 (0.71, 1.60)	-6.03 (-6.79, -5.27)	8.34 (7.58, 9.10)
**16–17**	WC1	283	1.79 (1.51, 2.08)	-2.99 (-3.48, -2.50)	6.57 (6.09, 7.06)	278	2.97 (2.64, 3.30)	-2.46 (-3.02, -1.90)	8.40 (7.84, 8.96)
	WC2	283	-0.22 (-0.62, 0.17)	-6.83 (-7.51, -6.16)	6.39 (5.71, 7.06)	278	-0.54 (-1.04, -0.04)	-8.82 (-9.67, -7.96)	7.73 (6.88, 8.58)
	WC3	283	0.63 (0.23, 1.03)	-6.04 (-6.73, -5.36)	7.31 (6.63, 7.99)	278	1.29 (0.76, 1.83)	-7.62 (-8.53–6.70)	10.20 (9.29, 11.12)

WC1: Waist circumference (WC) measured at umbilicus

WC2: WC measured immediately above the iliac crest

WC3: WC measured at midpoint of the lower margin of the last palpable rib and top of the iliac crest

Bias: Mean difference

95%CI: 95% confidence interval

LLOA: Lower limits of agreement

ULOA: Upper limits of agreement

N: Sample size in the subgroup

The LOA results indicated that the degree of between-measure agreement for each WC measurement site was within ±95% LOA in 96% of measurements.

#### Waist circumference measured at umbilicus (WC1)

Among the boys, the assessor-measured WC values at WC1 were on average larger than the self-measured WC values (MD: 0.3 [SD: 4.2] to 1.8 [2.8]} regardless of age. There were no statistically significant mean differences between assessor-measured WC and self-measured WC except for those aged 12 to 13 (1.8 [2.8], p = 0.03) and 16 to 17 (1.8 [2.4], p = 0.03) years old. The narrowest LOA (-2.61, 5.08) was found in boys aged 10 to 11.

The results for the girls were similar to those for the boys. The assessor-measured WC values at this site were on average larger than the self-measured WC values (0.5 [3.7] to 3.3 [3.9]) regardless of age. With the exception of the results for those aged 6 to 9 years old, the mean differences between assessor-measured WC and self-measured WC groups was found statistically significant different in girls aged 10 to 17 (1.9 [2.7], p<0.0001 to 3.0 [2.8], p<0.0001). The narrowest LOA width (-3.43, 7.13) was found in girls aged 10 to 11.

#### Waist circumference measured immediately above the iliac crest (WC2)

Across the age groups for boys, the assessor-measured WC values at WC2 were on average smaller than the self-measured WC values at ages 6 to 9 (-1.0 [5.1] to -1.0 [4.2]) and 14 to 17 (-0.1 [3.5] to -0.2 [3.4]). For boys aged 10 to 13, however, the reverse was true (0.00 [2.9] to 0.1 [3.4]). The mean differences between assessor-measured WC and self-measured WC did not differ significantly in all age groups. The narrowest LOA (-5.61, 5.65) was found at ages 10 to 11.

In girls, assessor-measurements on average were smaller than self-measurements at ages 6 to 9 (-1.6 [4.9] to -0.8 [4.0]) and 16 to 17 (-0.5 [4.2]). Among those aged 10 to 15 years old, in contrast, the reverse was true (0.1 [3.8] to 0.0 [4.0]). The mean differences between assessor-measured WC and self-measured WC did not differ significantly at all ages except for the 6 to 7 years old (1.6 [4.9, p = 0.03]). The narrowest LOA (-7.44, 7.62) was found in girls aged 10 to 11.

#### Waist circumference measured at the midpoint of the lower margin of the last palpable rib and top of the iliac crest (WC3)

Finally, among boys, the assessor-measured WC values at WC3 were on average smaller than the self-measured WC values at ages 6 to 11 (-0.2 [3.7] to -0.2 [2.6]). For those aged 12 and above, the reverse was true (0.3[4.0] to 0.6[3.4]). The mean differences between assessor-measured WC and self-measured WC did not differ significantly at all ages. The narrowest LOA widths (-4.26, 5.19) was found at ages 14 to 15.

Among girls, the assessor-measured WC values were on average smaller than the self-measured WC at ages 6 to 9 (-0.6 [4.2] to -0.4 [4.6]), whereas the opposite result was found for those aged 10 and above (0.5 [3.1] to 1.3 [0.45]). The assessor-measured mean difference was significantly different from that of the self-measurements at age 16 to 17 (1.3 [4.5, p = 0.04]). The narrowest LOA widths (-5.68, 6.59) was observed at ages 10 to 11.

#### Waist circumference measurements among obese children/adolescents

Among obese children/adolescents, the largest mean difference between assessor-measured and self-measured WC was found at WC1 in girls (3.3 [SD: 3.3], in boys (2.0 [SD: 3.5] and at WC2 in boys (2.0 [SD: 4.8]) (S1, S2 & S3 Tables). We stratified the WC measurements at the three sites by weight status (normal, underweight, overweight, obese) and locations (Table 3). The results were similar to the age-group and sex-specific Bland-Altman level of agreement analysis (Table 2).

**Table 3:** Limits of agreement analysis of 3 waist circumference measurement sites between assessor-measured and home measured/self-measured values by weight status and locations in boys and girls.

	Boys						Girls				
Weight status	Location	N	Bias (95% CI)	LLOA (95% CI)	ULOA (95%CI)	Range of agreement	N	Bias (95% CI)	LLOA (95% CI)	ULOA (95% CI)	Range of agreement
**underweight**	WC1	29	0.44 (-0.57, 1.45)	-4.79 (-6.54, -3.04)	5.67 (3.92, 7.42)	10.46	33	2.26 (1.36, 3.15)	-2.71 (-4.26, -1.16)	7.23 (5.68, 8.78)	9.94
**normal**	WC1	1158	1.27 (1.12, 1.41)	-3.73 (-3.98, -3.48)	6.26 (6.01, 6.51)	9.99	987	2.28 (2.07, 2.49)	-4.29 (-4.65, -3.94)	8.85 (8.49, 9.21)	13.14
**Overweight**	WC1	228	1.34 (0.94, 1.74)	-4.66 (-5.34, -3.97)	7.34 (6.66, 8.03)	12.00	211	1.96 (1.58, 2.34)	-3.59 (-4.24, -2.93)	7.51 (6.85, 8.16)	11.10
**Obese**	WC1	201	2.00 (1.51, 2.5)	-4.94 (-5.78, -4.09)	8.95 (8.10, 9.79)	13.89	133	3.28 (2.72, 3.85)	-3.17 (-4.14, -2.20)	9.74 (8.77, 10.71)	12.91
**underweight**	WC2	29	-2.29 (-3.71, -0.88)	-9.60 (-12.05, -7.15)	5.01 (2.57, 7.46)	14.61	33	-1.46 (-3.22, 0.30)	-11.2 (-14.24, -8.16)	8.28 (5.24, 11.32)	19.48
**normal**	WC2	1158	-0.73 (-0.93, -0.54)	-7.29 (-7.62, -6.96)	5.83 (5.50, 6.16)	13.12	987	-0.47 (-0.72, -0.21)	-8.46 (-8.90, -8.03)	7.52 (7.09, 7.96)	15.98
**Overweight**	WC2	228	0.22 (-0.24, 0.68)	-6.67 (-7.45, -5.88)	7.10 (6.32, 7.89)	13.77	211	-0.39 (-0.9, 0.12)	-7.76 (-8.64, -6.89)	6.99 (6.11, 7.86)	14.75
**Obese**	WC2	201	2.00 (1.33, 2.67)	-7.42 (-8.56, -6.28)	11.42 (10.27, 12.56)	18.84	133	1.32 (0.46, 2.17)	-8.47 (-9.93, -7.00)	11.1 (9.63, 12.57)	19.57
**underweight**	WC3	29	0.00 (-0.93, 0.92)	-4.78 (-6.39, -3.18)	4.78 (3.18, 6.38)	9.56	33	-0.22 (-2.2, 1.75)	-11.17 (-14.59, -7.76)	10.72 (7.31, 14.14)	21.89
**normal**	WC3	1158	0.05 (-0.12, 0.23)	-5.99 (-6.29, -5.69)	6.10 (5.79, 6.40)	12.09	987	0.61 (0.38, 0.85)	-6.84 (-7.25, -6.44)	8.07 (7.66, 8.47)	14.91
**Overweight**	WC3	228	0.20 (-0.37, 0.77)	-8.32 (-9.29, -7.35)	8.72 (7.75, 9.69)	17.04	211	0.05 (-0.47, 0.57)	-7.52 (-8.42, -6.63)	7.62 (6.73, 8.52)	15.14
**Obese**	WC3	201	0.58 (0.03, 1.13)	-7.14 (-8.07, -6.20)	8.30 (7.36, 9.23)	15.44	133	1.68 (0.91, 2.44)	-7.12 (-8.43, -5.80)	10.47 (9.15, 11.78)	17.59

WC1: Waist circumference (WC) measured at umbilicus

WC2: WC measured immediately above the iliac crest

WC3: WC measured at midpoint of the lower margin of the last palpable rib and top of the iliac crest

Bias: Mean difference

95%CI: 95% confidence interval

LLOA: Lower limits of agreement

ULOA: Upper limits of agreement

N: Sample size in the subgroup

#### The reliability of measurements

Analysis of the reliability of measurements between assessor-measured WC and self-measured WC for each WC measurement site yielded ICC values ranging from 0.67 to 0.98 at all sites (S1, S2 & S3 Tables).

## Discussion

The aim of the study reported herein was to evaluate the validity of WC self-measurements at three WC sites taken by novice laypersons, including children/adolescents and/or their parents/guardians/carers, at home against that of WC measurements taken by trained assessors using a non-elastic sewing tape measure in accordance with standard measurement instructions. Bland-Altman statistical method was used to quantify agreement between two WC measurements by studying the mean difference and constructing limits of agreement [20]. The results of Bland-Altman analysis revealed a wide range of agreement exceeding the a priori defined difference criterion of ≤ 2.5 cm for the ULOA and LLOA, suggesting disagreement between the two WC measurement methods at all three sites.

The correlation between the self-measurements and their corresponding assessor measurements ranged from 0.67 to 0.98 at all three WC sites suggesting moderate to excellent reliability [21]. However, ICC statistics demonstrate the reliability and strength of the relationship between two raters; they do not reflect the inter-rater agreement and consider the measurement error. Measurement error assesses the value difference between repeated measurements within individuals [12]. Hence, ICC use can produce misleading results when assessing agreement using comparison data [17].

We carried out an inter-rater agreement test between the assessors and investigator to ensure a high degree of accuracy in measuring WC at the three sites. Two-sample t-tests revealed no significant mean differences between assessor-measured WC and investigator-measured WC for any of the measurements. As there is no guideline/reference for the interval of agreement in WC measurement, the a priori criterion of LOA was defined as ≤ 2.5 cm at both the ULOA and LLOA using the inter-rater agreement test results. We consider the range of LOA to be clinically acceptable for WC measurement at the three WC sites. Several prior studies [22–24] have validated self-measured WC against assessor-measured WC in adults without defining the a priori criterion or considering the ULOA and LLOA widths. Their validation results are thus open to question, as they report a wide range of LOA. Our study results provide evidence of interobserver WC measurement error (a priori criterion of LOA ≤ ± 2.5 cm) for clinicians and researchers to detect clinically relevant change of individual subjects over time [12].

Of the three WC sites, the umbilicus (WC1) is the easiest to locate. However, as it also has the greatest extension of abdominal fat and lacks a bony landmark, it has only one point to guide the measurement. In addition, measuring WC at this site on a plane that is perpendicular to the long body axis may be difficult, particularly for children/adolescents with a thick layer of abdominal fat or umbilicus that is located below the level of the iliac crest, which is the case in very obese children/adolescents. Therefore, the measurement at WC1 may not be a true measure of where the umbilicus anatomically situated in normal weight and underweight children/adolescent. This may provide an explanation for the largest mean differences at this site between the self-measured WC and assessor-measured WC for both sexes in obese children/adolescents. These results imply that using soft tissue without a bony landmark as a measurement guide results in greater variation between self and assessor measurements.

Measurements taken at WC2 and WC3 require both knowledge and skill to palpate the bony landmarks. Palpation to locate these landmarks may be difficult for individuals with limited anatomical knowledge of the top of the hip bone and lowest palpable rib. It may also be uncomfortable, particularly for obese children/adolescents who need to identify the correct location under a thick layer of fat [25]. A protocol that uses fixed skeletal anatomic landmarks to locate the measurement sites allows participants to obtain relatively reliable WC measurement results [26, 27] compared with measurement at the umbilicus (WC1), as such measurement is unaffected by changing levels of adiposity [27]. This is particularly important when repeat measurements are required.

Among all children/adolescents, the largest variation of mean differences between assessor-measured WC and self/home-measured WC were found in obese children/adolescents compared to normal weight and underweight children/adolescents regardless sex and age groups. This may due to difficulty in locating anatomical landmarks at the 3 WC measurement sites [28, 29]. Except for the age groups 6 to 7 years at WC2 in girls, there were no significant mean differences between assessor-measured WC and home-measured WC at all measurement sites in both sex. We reviewed the abdominal obesity rate in these age groups (6–9 years) and found a small percentage (4.8%) to exhibit such obesity. As the typical body shape in children of these ages without abdominal obesity is cylindrical, the parents may have been able to obtain results relatively close to those of the assessors, which may explain the lack of significant mean differences between assessor-measured WC and home-measured WC in these sites.

We provided participants with instructions for each WC measurement site. According to our observations, some participants were unable to keep their backs as straight and posture as upright as required when taking their own measurements. Some were also unable to ensure that the tape measure was perpendicular to their long body axis, and others failed to keep the tape measure flat and placed it at incorrect locations on the waist. In addition, because of the sharply curved skin surface superior to the iliac crest in some female adolescents, these participants often found it more difficult to stabilize the tape measure at this site relative to the two other sites, which may have resulted in measurement errors.

The assessment of abdominal obesity via WC measurement is of great importance, as it provides a measure of fat distribution and abdominal girth that cannot be obtained from individuals’ BMI. The former is also a very cost-effective way to identify abdominal obesity, particularly in individuals who have a low BMI but a big belly. The self-/home-measurement of WC in adolescents/children could be of importance in the self-monitoring of abdominal obesity in community settings and in epidemiological research. However, our study demonstrates wide LOA, suggesting disagreement between assessor-measured and self-/home-measured WC. Measurement should thus be conducted by individuals who have received proper training in self-measured WC. Both self-measured WC training using a video [30] and computer-based self-measured WC training [23] have been found to facilitate accurate WC measurements.

### Limitations

This study involved a cross-sectional convenience sample recruited from schools rather than a random sample because it is difficult to obtain the consent of school principals and parents for research that involves exposure of the midriff, as is necessary for WC measurements. This may reflect cultural reticence in part of the Hong Kong Chinese community. To minimize sampling bias, we recruited children/adolescents from the three main regions of Hong Kong: Hong Kong Island, Kowloon, and the New Territories. Another potential limitation is that more than one assessor (n = 6) performed the WC measurements, which may have introduced some degree of measurement bias. However, to minimize such bias, all six assessors took part in a WC measurement training session, and we tested the inter-rater agreement among assessors. We obtained good between-rater LOA, with no statistically significant mean differences found at any of the WC sites.

## Conclusions

The range of LOA in this study was wide, exceeding the acceptable limits of the predefined a priori criterion and thus suggesting disagreement between the two measurement methods, that is, self-measured WC and assessor-measured WC. Self-measured WC at all three sites may not be adequately sensitive for assessing and monitoring abdominal obesity, and changes therein, in children/adolescents. Hence, the use of WC to measure abdominal obesity in epidemiological studies or clinical practice should be restricted to measurement by trained health professionals/research staff because of the disagreement of self/home measurements.

## Supporting information

S1 Table Mean differences and intra-class correlations of waist circumference measured at umbilicus (WC1) between assessor-measured and home-measured/self-measured values by age group and weight status in boys and girls. (DOCX)

S2 Table Mean differences and intra-class correlations of waist circumference measured at immediately above the iliac crest (WC2) between assessor-measured and home-measured/self measured values by age group and weight status in boys and girls. (DOCX)

S3 Table Mean differences and intra-class correlations of waist circumference measured at midpoint between the lower margin of the last palpable rib and the top of the iliac crest (WC3) between assessor-measured and home-measured/self-measured values by gender, age group and weight status in boys and girls. (DOCX)

## References

[1] Student Health Service, Department of Health, The Hong Kong SAR. Health watch: healthy weight healthy kids. 2014. http://www.change4health.gov.hk/en/whats_new/health_watch/index_id_32.html.

[2] Mensah G, Mokdad A, Ford E, Narayan K, Giles W, Vinicor F, et al Obesity, metabolic syndrome, and type 2 diabetes: emerging epidemics and their cardiovascular implications. Cardiology Clinics. 2004;22(4):485–504. 10.1016/j.ccl.2004.06.005 15501618

[3] Basterfield L, Pearce MS, Adamson AJ, Reilly JK, Parkinson KN, Reilly JJ. Effect of choice of outcome measure on studies of the etiology of obesity in children. Annals of epidemiology. 2012;22(12):888–91. 10.1016/j.annepidem.2012.09.007 23084839

[4] World Health Organization. STEPS manual [Internet] Geneva, World Health Organization 2008. http://www.who.int/chp/steps/manual/en/index.html

[5] National Institutes of Health, National Heart Lung and Blood Institute, North American Association for the Study of Obesity. The practical guide to the identification, evaluation and treatment of overweight and obesity in adults. 2000 NIH Publication Number 00–4084.

[6] Klein S, Allison DB, Heymsfield SB, Kelley DE, Leibel RL, Nonas C, et al Waist circumference and cardiometabolic risk: a consensus statement from shaping America’s Health: Association for Weight Management and Obesity Prevention; NAASO, The Obesity Society; the American Society for Nutrition; and the American Diabetes Association. The American Journal of Clinical Nutrition. 2007;85(5):1197–202. 17490953 10.1093/ajcn/85.5.1197

[7] Pischon T, Boeing H, Hoffmann K, Bergmann M, Schulze MB, Overvad K, et al General and Abdominal Adiposity and Risk of Death in Europe. New England Journal of Medicine. 2008;359(20):2105–20. 10.1056/NEJMoa0801891 19005195

[8] Yusuf S, Hawken S, Ôunpuu S, Bautista L, Franzosi M, Commerford P, et al Obesity and the risk of myocardial infarction in 27 000 participants from 52 countries: a case-control study. The Lancet. 2005;366(9497):1640–9. 10.1016/S0140-6736(05)67663-5. 16271645

[9] Hara K, Matsushita Y, Horikoshi M, Yoshiike N, Yokoyama T, Tanaka H, et al A Proposal for the cutoff point of waist circumference for the diagnosis of metabolic syndrome in the Japanese Population. Diabetes Care. 2006;29(5):1123–4. 10.2337/diacare.2951123 16644651

[10] Chan NP, Choi KC, Nelson EA, Sung RY, Chan JC, Kong AP. Self-reported waist circumference: a screening tool for classifying children with overweight/obesity and cardiometabolic risk factor clustering. Pediatric Obesity. 2012;7(2):110–20. Epub 2012 Feb 10. 10.1111/j.2047-6310.2011.00017.x 22434750

[11] Ulijaszek S, Kerr D. Anthropometric measurement error and the assessment of nutritional status 1999 165–77 p. 10.1017/s0007114599001348 10655963

[12] Verweij LM, Terwee CB, Proper KI, Hulshof CT, van Mechelen W. Measurement error of waist circumference: gaps in knowledge. Public health nutrition. 2013;16(2):281–8. Epub 2012/05/26. 10.1017/S1368980012002741 . 22626254 PMC10271771

[13] Daly L, Bourke G. Interpretation and use of medical statistics. Oxford: Blackwell Science Ltd; 2000.

[14] Leung S, Cole T, Tse L, Lau J. Body mass index reference curves for Chinese children. Annals of Human Biology. 1998;25(2):169–74. 10.1080/03014469800005542 9533516

[15] Ng V, Kong A, Choi K, Ozaki R, Wong G, So W, et al BMI and waist circumference in predicting cardiovascular risk factor clustering in Chinese adolescents. Obesity. 2007;15:494–503. 10.1038/oby.2007.588 17299123

[16] Sung R, So H, Choi K, Nelson E, Li A, Yin J, et al Waist circumference and waist-to-height ratio of Hong Kong Chinese children. BMC Public Health. 2008;8(1):324. 18808684 10.1186/1471-2458-8-324 PMC2563004

[17] Bland JM, Altman DG. Applying the right statistics: analyses of measurement studies. Ultrasound in obstetrics & gynecology: the official journal of the International Society of Ultrasound in Obstetrics and Gynecology. 2003;22(1):85–93. Epub 2003/07/15. 10.1002/uog.122 . 12858311

[18] British Standards Institution. Precision of test methods 1: Guide for the determination and reproducibility for a standard test method (BS 597, Part 1). London: BSI; 1975.

[19] Mantha S, Roizen MF, Fleisher LA, Thisted R, Foss J. Comparing methods of clinical measurement: reporting standards for bland and altman analysis. Anesthesia and analgesia. 2000;90(3):593–602. Epub 2000/03/07. . 10702443 10.1097/00000539-200003000-00018

[20] Giavarina D. Understanding Bland Altman analysis. Biochemia Medica. 2015;25(2):141–51 26110027 10.11613/BM.2015.015 PMC4470095

[21] Portney L, Watkins M. Foundations of clinical research: applications to practice. New Jersey: Prentice Hall; 2000.

[22] Barrios P, Martin-Biggers J, Quick V, Byrd-Bredbenner C. Reliability and criterion validity of self-measured waist, hip, and neck circumferences. BMC Medical Research Methodology. 2016;16(1):1–12. 10.1186/s12874-016-0150-2 27145829 PMC4855335

[23] Elliott WL. Criterion validity of a computer-based tutorial for teaching waist circumference self-measurement. Journal of Bodywork and Movement Therapies. 2008;12(2):133–45. 10.1016/j.jbmt.2007.10.007 19083665

[24] Contardo Ayala AM, Nijpels G, Lakerveld J. Validity of self-measured waist circumference in adults at risk of type 2 diabetes and cardiovascular disease. BMC Medicine. 2014;12:170 10.1186/s12916-014-0170-x 25274418 PMC4192531

[25] Rudolf M, Walker J, Cole T. What is the best way to measure waist circumference? International Journal of Pediatric Obesity. 2007;2(1):58–61 17763011 10.1080/17477160601095177

[26] Ross R, Berentzen T, Bradshaw A, Janssen I, Kahn H, Katzmarzyk P, et al Does the relationship between waist circumference, morbidity and mortality depend on measurement protocol for waist circumference? Obesity Reviews. 2008;9(4):312–25. 10.1111/j.1467-789X.2007.00411.x 17956544

[27] Mason C, Katzmarzyk PT. Variability in waist circumference measurements according to anatomic measurement site. Obesity (Silver Spring). 2009;17(9):1789–95. Epub 2009/04/04. 10.1038/oby.2009.87 . 19343017

[28] Agarwal SK, Misra A, Aggarwal P, Bardia A, Goel R, Vikram NK, et al Waist circumference measurement by site, posture, respiratory phase, and meal time: implications for methodology. Obesity (Silver Spring, Md). 2009;17(5):1056–61. 10.1038/oby.2008.635 19165166

[29] Wang CY, Liu MH, Chen YC. Intrarater reliability and the value of real change for waist and hip circumference measures by a novice rater. Perceptual and motor skills. 2010;110(3 Pt 2):1053–8. Epub 2010/09/28. . 20865992 10.2466/pms.110.C.1053-1058

[30] McEneaney DF, Lennie SC. Video instructions improve accuracy of self-measures of waist circumference compared with written instructions. Public health nutrition. 2011;14(7):1192–9. Epub 2011/04/01. 10.1017/S1368980011000450 . 21450137

