# Supplementary material for: Agreement between self-/home-measured and assessor-measured waist circumference at three sites in adolescents/children
Source: PLoS One. 2018 Mar 22;13(3):e0193355. doi: 10.1371/journal.pone.0193355 (PMC5863965; doi:10.1371/journal.pone.0193355)
Supplement: S1 Table — (DOCX) [file pone.0193355.s001.docx]

**S1 Table A** Mean differences and intra-class correlations of waist circumference measured at umbilicus (WC1) between

assessor-measured and home-measured/self-measured values by age group and weight status in boys and girls

| **Sex** | **Age group**  **(in years)** | **Mean of assessor-measured WC**  **(SD) in cm** | **Mean of parent-/self-measured WC (SD) in cm.** | **Mean**  **Difference (SD)** | **^†^p-value** | **^Ψ^ICC (95% CI)** |
| --- | --- | --- | --- | --- | --- | --- |
| All | (n=2980) | 68.7 (11.3) | 66.9 (10.9) | 1.8 (3.1) | <0.0001 | 0.949 (0.946, 0.953) |
|  |  |  |  |  |  |  |
| Boys | All (n=1616) | 69.4 (12.3) | 68.0 (12.0) | 1.4 (2.8) | <0.0001 | 0.968 (0.965, 0.971) |
| Girls | All (n=1364) | 67.9 (9.8) | 65.5 (9.3) | 2.3 (3.3) | <0.0001 | 0.914 (0.904, 0.922) |
|  | **Assessor-measured and home-measured WC at WC1** | | | | | |
| Boys | 6 - 7 (n=204) | 56.2 (7.2) | 55.9 (7.7) | 0.3 (4.2) | 0.72 | 0.846 (0.803, 0.881) |
|  | 8 - 9 (n=216) | 62.0 (9.1) | 61.1 (8.8) | 0.9 (2.6) | 0.30 | 0.952 (0.938, 0.963) |
|  |  |  |  |  |  |  |
| Girls | 6 - 7 (n=155) | 55.6 (6.0) | 55.1 (6.3) | 0.5 (3.7) | 0.46 | 0.820 (0.761, 0.865) |
|  | 8 - 9 (n=183) | 61.7 (8.5) | 60.4 (8.4) | 1.2 (2.9) | 0.16 | 0.931 (0.909, 0.948) |
|  | **Assessor-measured and self-measured WC at WC1** | | | | | |
| Boys | 10 - 11 (n=248) | 68.5 (10.9) | 67.3 (10.9) | 1.2 (2.0) | 0.21 | 0.978 (0.971, 0.983) |
|  | 12 - 13 (n=369) | 72.2 (11.2) | 70.4 (11.4) | 1.8 (2.8) | 0.03 | 0.958 (0.949, 0.966) |
|  | 14 - 15 (n=296) | 75.4 (12) | 73.8 (12.0) | 1.6 (2.3) | 0.12 | 0.973 (0.966, 0.978) |
|  | 16 - 17 (n=283) | 75.2 (9.8) | 73.4 (9.4) | 1.8 (2.4) | 0.03 | 0.951 (0.938, 0.961) |
|  |  |  |  |  |  |  |
| Girls | 10 - 11 (n=239) | 66.5 (8.8) | 64.7 (9.0) | 1.9 (2.7) | 0.02 | 0.934 (0.916, 0.948) |
|  | 12 - 13 (n=245) | 70.9 (9.0) | 68.0 (8.6) | 3.0 (2.8) | <0.0001 | 0.894 (0.866, 0.917) |
|  | 14 - 15 (n=264) | 72.0 (7.5) | 68.8 (7.5) | 3.3 (3.9) | <0.0001 | 0.782 (0.731, 0.825) |
|  | 16 - 17 (n=278) | 73.2 (7.2) | 70.3 (7.3) | 3.0 (2.8) | <0.0001 | 0.851 (0.815, 0.880) |
|  | **Weight Status** |  |  |  |  |  |
| Boys | Underweight (n=29) | 55.9 (5.9) | 55.5 (5.4) | 0.4 (2.7) | 0.77 | 0.890 (0.781, 0.946) |
|  | Normal (n=1158) | 64.7 (8.0) | 63.5 (7.8) | 1.3 (2.5) | <0.0001 | 0.936 (0.928, 0.942) |
|  | Overweight (n=228) | 76.7 (9.3) | 75.4 (8.9) | 1.3 (3.1) | 0.12 | 0.934 (0.915, 0.948) |
|  | Obese (n=201) | 89.6 (11.2) | 87.6 (11.6) | 2.0 (3.5) | 0.08 | 0.937 (0.917, 0.952) |
|  |  |  |  |  |  |  |
| Girls | Underweight (n=33) | 56.9 (5.6) | 54.7 (3.9) | 2.3 (2.5) | 0.06 | 0.773 (0.590, 0.881) |
|  | Normal (n=987) | 65.0 (7.9) | 62.8 (7.2) | 2.3 (3.4) | <0.0001 | 0.860 (0.843, 0.876) |
|  | Overweight (n=211) | 73.5 (7.4) | 71.5 (7.4) | 2.0 (2.8) | 0.01 | 0.894 (0.864, 0.918) |
|  | Obese (n=133) | 82.6 (8.9) | 79.3 (8.9) | 3.3 (3.3) | <0.0001 | 0.868 (0.819, 0.905) |
|  |  |  |  |  |  |  |

Mean difference : mean of assessor-measured minus mean of parent-measured/self-measured.

SD : standard deviation

^Ψ^ICC: Intra-cl ass correlation coefficient.

**^†^**p-value : ^†^Two sample student's *t* -test.

95%CI : 95% confidence interval.
