# Supplementary material for: Agreement between self-/home-measured and assessor-measured waist circumference at three sites in adolescents/children
Source: PLoS One. 2018 Mar 22;13(3):e0193355. doi: 10.1371/journal.pone.0193355 (PMC5863965; doi:10.1371/journal.pone.0193355)
Supplement: S2 Table — (DOCX) [file pone.0193355.s002.docx]

**S2 Table B** Mean differences and intra-class correlations of waist circumference measured at immediately above the

iliac crest (WC2) between assessor-measured and home-measured/self-measured values by age group and weight status in

boys and girls

| **Sex** | **Age group**  **(in years)** | **Mean of assessor-measured WC**  **(SD) in cm** | **Mean of parent-/self-measured WC (SD) in cm.** | **Mean Difference (SD)** | **^†^p-value** | ^Ψ^**ICC (95% CI)** |
| --- | --- | --- | --- | --- | --- | --- |
| All | (n=2980) | 69.1 (11.3) | 69.4 (10.9) | -0.3 (3.9) | 0.30 | 0.937 (0.933, 0.941) |
|  |  |  |  |  |  |  |
| Boys | All (n=1616) | 69.3 (12.1) | 69.6 (11.6) | -0.3 (3.7) | 0.49 | 0.951 (0.946, 0.955) |
| Girls | All (n=1364) | 68.9 (10.3) | 69.2 (10.1) | -0.3 (4.2) | 0.43 | 0.916 (0.907, 0.924) |
|  | **Assessor-measured and home-measured WC at WC2** | | | | | |
| Boys | 6 - 7 (n=204) | 56.3 (7.3) | 57.3 (7.6) | -1.0 (5.1) | 0.18 | 0.760 (0.696, 0.813) |
|  | 8 - 9 (n=216) | 61.8 (9.0) | 62.8 (8.8) | -1.0 (4.2) | 0.25 | 0.884 (0.851, 0.910) |
|  |  |  |  |  |  |  |
| Girls | 6 - 7 (n=155) | 55.5 (5.8) | 57.1 (6.7) | -1.6 (4.9) | 0.03 | 0.672 (0.576, 0.750) |
|  | 8 - 9 (n=183) | 61.7 (8.4) | 62.6 (8.4) | -0.8 (4.0) | 0.35 | 0.884 (0.848, 0.912) |
|  | **Assessor-measured and self-measured WC at WC2** | | | | | |
| Boys | 10 - 11 (n=248) | 68.1 (10.7) | 68.1 (9.9) | 0.0 (2.9) | 0.98 | 0.961 (0.950, 0.970) |
|  | 12 - 13 (n=369) | 72.1 (11.0) | 72 (10.8) | 0.1 (3.4) | 0.90 | 0.953 (0.942, 0.961) |
|  | 14 - 15 (n=296) | 75.4 (11.3) | 75.5 (10.9) | -0.1 (3.5) | 0.91 | 0.952 (0.940, 0.962) |
|  | 16 - 17 (n=283) | 75.4 (9.6) | 75.6 (8.6) | -0.2 (3.4) | 0.77 | 0.931 (0.914, 0.945) |
|  |  |  |  |  |  |  |
| Girls | 10 - 11 (n=239) | 67.0 (8.9) | 66.9 (9.3) | 0.1 (3.8) | 0.91 | 0.912 (0.887, 0.931) |
|  | 12 - 13 (n=245) | 72.2 (8.9) | 71.8 (9.0) | 0.4 (4.1) | 0.63 | 0.893 (0.865, 0.916) |
|  | 14 - 15 (n=264) | 73.6 (7.7) | 73.6 (7.5) | 0.0 (4.0) | 0.97 | 0.861 (0.826, 0.889) |
|  | 16 - 17 (n=278) | 75.1 (7.7) | 75.7 (7.2) | -0.5 (4.2) | 0.39 | 0.839 (0.801, 0.871) |
|  | **Weight Status** |  |  |  |  |  |
| Boys | Underweight (n=29) | 56.2 (6.1) | 58.5 (6.5) | -2.3 (3.7) | 0.17 | 0.773 (0.574, 0.886) |
|  | Normal (n=1158) | 64.8 (8) | 65.5 (8.1) | -0.7 (3.3) | 0.03 | 0.910 (0.900, 0.919) |
|  | Overweight (n=228) | 76.5 (9.1) | 76.3 (9.1) | 0.2 (3.5) | 0.80 | 0.926 (0.904, 0.942) |
|  | Obese (n=201) | 89.0 (10.8) | 87.0 (11.4) | 2.0 (4.8) | 0.07 | 0.892 (0.860, 0.917) |
|  |  |  |  |  |  |  |
| Girls | Underweight (n=33) | 58.3 (6.7) | 59.8 (5.8) | -1.5 (5) | 0.35 | 0.671 (0.432, 0.822) |
|  | Normal (n=987) | 66.1 (8.6) | 66.6 (8.7) | -0.5 (4.1) | 0.23 | 0.888 (0.874, 0.900) |
|  | Overweight (n=211) | 74.2 (8.0) | 74.6 (8.1) | -0.4 (3.8) | 0.62 | 0.891 (0.859, 0.916) |
|  | Obese (n=133) | 83.4 (9.8) | 82.0 (9.8) | 1.3 (5.0) | 0.27 | 0.862 (0.811, 0.900) |
|  |  |  |  |  |  |  |

Mean difference : mean of assessor-measured minus mean of parent-measured/self-measured.

SD : standard deviation

^Ψ^ICC: Intra-cl ass correlation coefficient.

**^†^**p-value : ^†^Two sample student's *t* -test.

95%CI : 95% confidence interval.
