# Supplementary material for: Agreement between self-/home-measured and assessor-measured waist circumference at three sites in adolescents/children
Source: PLoS One. 2018 Mar 22;13(3):e0193355. doi: 10.1371/journal.pone.0193355 (PMC5863965; doi:10.1371/journal.pone.0193355)
Supplement: S3 Table — (DOCX) [file pone.0193355.s003.docx]

**S3 Table C** Mean differences and intra-class correlations of waist circumference measured at midpoint between the lower margin of the last palpable rib and the top of the iliac crest (WC3) between assessor-measured and home-measured/self-measured values by gender, age group and weight status in boys and girls

| **Sex** | **Age group**  **(in years)** | **Mean of assessor-measured WC (SD) in cm** | **Mean of parent-/self-measured WC (SD) in cm.** | **Mean**  **Difference (SD)** | **^†^p-value** | ^Ψ^**ICC (95% CI)** |
| --- | --- | --- | --- | --- | --- | --- |
| All | (n=2980) | 66.5 (10.7) | 66.1 (10.7) | 0.4 (3.7) | 0.20 | 0.940 (0.936, 0.944) |
|  |  |  |  |  |  |  |
| Boys | All (n=1616) | 67.6 (11.6) | 67.4 (11.7) | 0.1 (3.4) | 0.73 | 0.957 (0.953, 0.961) |
| Girls | All (n=1364) | 65.2 (9.2) | 64.6 (9.1) | 0.6 (4.0) | 0.08 | 0.905 (0.895, 0.914) |
|  | **Assessor-measured and home-measured WC at WC3** | | | | | |
| Boys | 6 - 7 (n=204) | 55.3 (6.8) | 55.6 (6.5) | -0.2 (3.7) | 0.70 | 0.842 (0.797, 0.878) |
|  | 8 - 9 (n=216) | 60.5 (8.7) | 61.1 (9.0) | -0.6 (3.7) | 0.49 | 0.913 (0.888, 0.933) |
|  |  |  |  |  |  |  |
| Girls | 6 - 7 (n=155) | 54.1 (5.6) | 54.6 (5.8) | -0.6 (4.2) | 0.36 | 0.719 (0.633, 0.787) |
|  | 8 - 9 (n=183) | 59.5 (8.0) | 59.9 (8.1) | -0.4 (4.6) | 0.64 | 0.838 (0.789, 0.876) |
|  | **Assessor-measured and self-measured WC at WC3** | | | | | |
| Boys | 10 - 11 (n=248) | 66.6 (10.4) | 66.8 (10.6) | -0.2 (2.6) | 0.87 | 0.969 (0.960, 0.976) |
|  | 12 - 13 (n=369) | 70.1 (10.6) | 69.7 (11.4) | 0.3 (4.0) | 0.68 | 0.934 (0.919, 0.946) |
|  | 14 - 15 (n=296) | 73.3 (11.2) | 72.9 (11.2) | 0.5 (2.4) | 0.61 | 0.976 (0.970, 0.981) |
|  | 16 - 17 (n=283) | 73.3 (9.2) | 72.6 (9.4) | 0.6 (3.4) | 0.42 | 0.931 (0.914, 0.945) |
|  |  |  |  |  |  |  |
| Girls | 10 - 11 (n=239) | 64.0 (8.4) | 63.6 (8.8) | 0.5 (3.1) | 0.56 | 0.933 (0.915, 0.948) |
|  | 12 - 13 (n=245) | 68.1 (8.2) | 67.2 (8.8) | 0.9 (3.2) | 0.24 | 0.925 (0.904, 0.941) |
|  | 14 - 15 (n=264) | 68.9 (6.9) | 67.8 (7.6) | 1.2 (3.7) | 0.07 | 0.860 (0.825, 0.888) |
|  | 16 - 17 (n=278) | 70.3 (7.6) | 69.0 (7.3) | 1.3 (4.5) | 0.04 | 0.799 (0.752, 0.838 |
|  | **Weight Status** |  |  |  |  |  |
| Boys | Underweight (n=29) | 55.0 (5.6) | 55.0 (5.5) | 0.0 (2.4) | 0.99 | 0.906 (0.812, 0.955) |
|  | Normal (n=1158) | 63.2 (7.5) | 63.2 (7.6) | 0.1 (3.1) | 0.86 | 0.917 (0.908, 0.926) |
|  | Overweight (n=228) | 74.3 (8.7) | 74.0 (9.4) | 0.2 (4.3) | 0.81 | 0.885 (0.853, 0.910) |
|  | Obese (n=201) | 86.7 (10.9) | 86.2 (11.3) | 0.6 (3.9) | 0.60 | 0.936 (0.916, 0.951) |
|  |  |  |  |  |  |  |
| Girls | Underweight (n=33) | 55.2 (5.6) | 55.5 (5.4) | -0.2 (5.6) | 0.87 | 0.496 (0.192, 0.714) |
|  | Normal (n=987) | 62.5 (7.3) | 61.9 (7.1) | 0.6 (3.8) | 0.06 | 0.856 (0.838, 0.872) |
|  | Overweight (n=211) | 70.4 (7.0) | 70.4 (7.6) | 0.0 (3.9) | 0.94 | 0.861 (0.821, 0.892) |
|  | Obese (n=133) | 79.3 (9.0) | 77.6 (9.3) | 1.7 (4.5) | 0.14 | 0.865 (0.815, 0.902) |

Mean difference : mean of assessor-measured minus mean of parent-measured/self-measured.

SD : standard deviation

^Ψ^ICC: Intra-cl ass correlation coefficient.

**^†^**p-value : ^†^Two sample student's *t* -test.

95%CI : 95% confidence interval.
